# Social Eavesdropping: Can You Hear the Emotionality in a “Hello” That Is Not Meant for You?

**DOI:** 10.1177/2041669517695816

**Published:** 2017-03-08

**Authors:** Sethu Karthikeyan, Vijayachandra Ramachandra

**Affiliations:** Pace University, NY, USA; Marywood University, Scranton, PA, USA

**Keywords:** emotion, multisensory or cross-modal processing, social cognition, speech, evolution, eavesdropping

## Abstract

The study examined third-party listeners’ ability to detect the Hellos spoken to prevalidated happy, neutral, and sad facial expressions. The average detection accuracies from the happy and sad (HS), happy and neutral (HN), and sad and neutral (SN) listening tests followed the average vocal pitch differences between the two sets of Hellos in each of the tests; HS and HN detection accuracies were above chance reflecting the significant pitch differences between the respective Hellos. The SN detection accuracy was at chance reflecting the lack of pitch difference between sad and neutral Hellos. As expected, the SN detection accuracy positively correlated with theory of mind; participating in these tests has been likened to the act of eavesdropping, which has been discussed from an evolutionary perspective. An unexpected negative correlation between the HS detection accuracy and the empathy quotient has been discussed with respect to autism research on empathy and pitch discrimination.

## Introduction

In a recent study, we employed a simulated help-seeking situation in which we had female participants ask photographs of a woman displaying expressions of happy, sad, and neutral emotions, for help with directions (Karthikeyan & Ramachandra, 2016). We expected that the emotional displays of the woman—the potential help-provider—would be mimicked vocally. This is because (a) mimickees tend to be prosocial toward mimickers (e.g., see Van Baaren, Holland, Steenaert, & Knippenberg, 2003, for prosociality as a result of verbal mimicry; see Van Baaren, Holland, Kawakami, & Van Knippenberg, 2004, for prosociality as a consequence of mimicry of body postures) and (b) emotional mimicry demonstrates cross-modal effects and is not restricted to the same modality, for example, corresponding facial expressions of emotions are triggered (as measured via electromyogram) while listening to others’ vocal emotions and not just while observing facial expressions (e.g., Magnee, Stekelenburg, Kemner, & de Gelder, 2006).

We found evidence for potential vocal mimicry in women’s Hellos directed to the three different facial emotional expressions (faces from hereon) in that the Hellos followed predictable pitch patterns (Karthikeyan & Ramachandra, 2016); those Hellos directed to the happy and sad faces and to the happy and neutral faces differed in average pitch—higher mean f_0_ or fundamental frequency in response to the happy face than to the sad and neutral faces—suggesting that the changes in average pitch followed visual cues of physiological arousal. In other words, there was a significant difference in f_0_ between Hellos directed to the happy and sad faces (happy and sad Hellos), and those spoken to the happy and neutral faces (happy and neutral Hellos), but there was no difference in f_0_ between Hellos directed to the sad and neutral faces (sad and neutral Hellos).

In the current study, we expected third-party listeners’ ability to detect emotionality in these Hellos to follow the differences in the average pitch—a salient acoustic cue. To reduce abstraction in the listening tests and facilitate detection of emotionality, we presented the photographs of the prevalidated facial expressions to listeners as they listened to the Hellos that were spoken to these faces. We hypothesized that when happy and sad Hellos are randomly presented to the participants (Happy–Sad or HS listening test), one at a time, along with the prevalidated happy and sad facial expressions, the accuracy of detection of these Hellos (HS detection accuracy) would be above chance. Similarly, when the happy and neutral Hellos are randomly presented to listeners (Happy–Neutral or HN listening test), one at a time, along with the prevalidated happy and neutral expressions, listeners will be able to detect these accurately at an above-chance level (HN detection accuracy). When sad and neutral Hellos are presented in the same format as above (Sad–Neutral or SN listening test), we expected only a chance or below-chance detection accuracy (SN detection accuracy), given that the average pitch of these Hellos did not differ (Karthikeyan & Ramachandra, 2016).

In the previous study, we also found that the standard deviations (SDf_0_s) or variability of pitch of the sad and neutral Hellos differed; additionally, the magnitude of this difference positively covaried with the speakers’ empathic capacity measured via the empathy quotient (EQ; Baron-Cohen & Wheelwright, 2004). This correlation with EQ was, however, not seen for the magnitude of pitch variability for happy versus sad and happy versus neutral Hellos. This was taken to suggest that the difference in the degree of vocal monotonousness to faces with different valence but with relatively similar arousal levels, specifically, to the sad and neutral faces (and not to the happy and sad or happy and neutral faces)^1^ was associated with the empathic capacity of speakers (Karthikeyan & Ramachandra, 2016). We hypothesized that in the same way as the speakers’ EQ correlated with the difference in pitch variability while greeting these faces, the SN detection accuracy may be associated with the listeners’ EQ. Empathic capacity, which involves the ability to internally simulate the perceived emotion (Oberman & Ramachandran, 2007), may influence the ability to identify an appropriately mimicked Hello in the SN listening test.

Empathy, however, consists of both cognitive empathy, also known as theory of mind (ToM) ability, and affective empathy. One can have unimpaired or superior mind reading skills despite poor or unrelated affective empathy as shown with psychopaths (Blair, 2007) and actors (Goldstein, Wu, & Winner, 2009). We therefore examined this measure of social sensitivity via the ToM test called the *Reading the mind in the eyes test* (Baron-Cohen, Wheelwright, Hill, Raste, & Plumb, 2001) in addition to EQ. The SN detection accuracy, like EQ, may be positively correlated with ToM ability.

Given that the vocal changes between sad and neutral Hellos may be indicative of how empathic the speaker is (Karthikeyan & Ramachandra, 2016), the SN listening test indirectly examines the extent to which participants are able to discern mental state information, empathy in this case, making it somewhat comparable to the ToM test; the main difference being the modality that is tested—visual in the latter (detailed in the Methods section). The possibility of an association between SN detection accuracy and ToM scores has evolutionary implications, as participating in these tests resembles the activity of eavesdropping i.e., observing or listening to others covertly, a phenomenon that has been discussed as a biological adaptation beneficial to social organisms in the context of group living (Locke, 2005, 2010). Trivers (1971), in his seminal paper on the evolution of reciprocal altruism, predicted that natural selection would favor such indirect forms of social learning in humans, as these would help in assessing the cooperative tendencies of others.

## Method

The institutional review board approved the study. Participants were students at a University in North America (*n* = 56; 28 women; age range: 18–35 years) who self-reported to be native speakers of American English and generally in good health with no sensory or neurological issues. They were either given course credit or $5 for participation. Each of the participants was subjected to three sets of listening experiments namely, HS, HN, or SN listening tests, each of which comprised the respective Hellos from 40 women speakers. Depending on the particular listening test, each of the participants, seated in a sound treated room, was presented with the respective Hellos (total stimuli per test = 80 Hellos, 40 of each of the two emotions in question),^2^ one at a time via headphones, in a random order, while also looking at the corresponding pair of facial expressions of emotions on a computer screen. The tests adopted a two-alternative forced choice response format and were presented via a macro-enabled PowerPoint presentation.

Consider one of the listening tests as an example—the HS test: In this test, 40 happy and 40 sad response Hellos were presented to the participant through headphones one at a time in a random order; with each Hello presentation (either happy or sad alone), the appropriate pair of photographs—in this case, the happy and sad expressions of the woman (prevalidated expressions from Lawrence & Abdel-Nabi, 2011)—were presented side by side on the computer screen. The side of presentation of the happy and sad faces was counterbalanced. The letters H (for happy) and S (for sad) appeared on top of these expressions as well.

Participants were told that they were looking at photographs of happy and sad expressions to which the Hellos they were about to hear were directed. Upon listening to each Hello, they were asked to select the expression to which they thought the Hello was spoken, by clicking on the accompanying letter (H or S). Each response click was automatically saved to an excel sheet in terms of correct or wrong, which the participants could not see. The response click would then lead to the next slide with the same pair of faces, but a different Hello (happy or sad), and so on. Percentages of the correct responses were automatically calculated (HS detection accuracy scores). The other two listening tests followed the same protocol. Participants went through a brief trial session in the beginning to familiarize themselves with the listening test. The experimenter left the room after providing the instructions in order to assure privacy.

After having completed all three listening tests, every participant completed the ToM and EQ tests. The ToM test (the *Reading the Mind in the Eyes test*) consists of 37 black and white images of the eye region including one practice item that display various expressions; every page of the test presents a single image with four words describing mental states (e.g., skeptical, panicked, jealous, and playful; Baron-Cohen et al., 2001). The participant is asked to choose the most appropriate word describing the expression. Also, a list containing the meaning of each of the words is included in the test packet. The EQ is a questionnaire comprising 60 items of which 40 are statements that examine empathy (e.g., I can pick up quickly if someone says one thing but means another), and 20 are control or filler items unrelated to empathy (e.g., I dream most nights; Baron-Cohen & Wheelwright, 2004). The EQ and ToM are both self-paced and self-report tests. The order of the three listening tests and that of the ToM and EQ tests was randomized for each of the participants.

## Results

**Figure 1.**
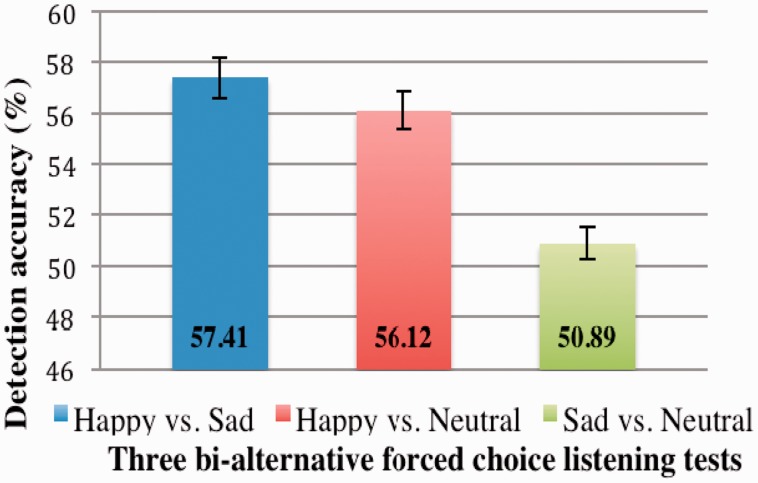
Detection accuracies obtained in happy and sad, happy and neutral, and sad and neutral listening tests.

As hypothesized, the detection patterns were in line with the average pitch differences (see Figure 1); the above-chance detection accuracies that HS and HN listening tests yielded (one-sample *t* tests, HS: *t*(55) = 9.29, HN: *t*(55) = 8.09; both *p* = .00) reflect the significant pitch differences between happy and sad f_0_s and happy and neutral f_0_s. As expected, the SN detection accuracy was not significantly above chance unlike the other two detection accuracies, (*t*(55) = 1.42, *p* = .08)^3^; recall that sad and neutral f_0_s were not different. This also indicates that the significant difference in pitch variability (SDf_0_s) between sad and neutral Hellos was not discernible.

**Figure 2.**
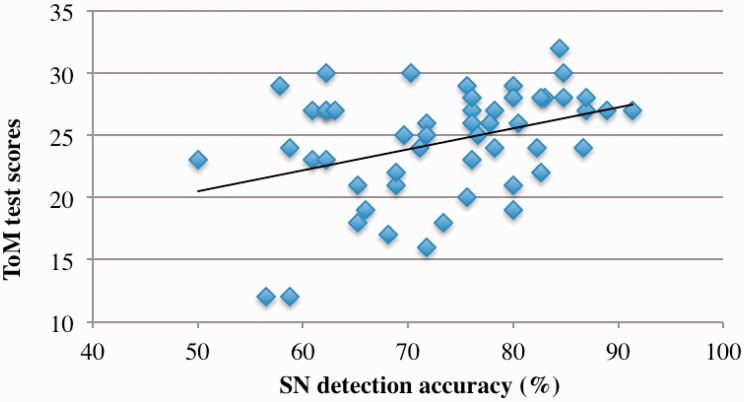
Positive correlation between the accuracy with which sad and neutral Hellos were detected in the sad and neutral listening test (sad and neutral detection accuracy) and the theory of mind test scores.

When the two outliers in the ToM scores (more than 2 *SD* away from the mean score) were removed, as hypothesized, a low, but statistically significant, positive correlation emerged between SN detection accuracy and ToM (*r* = 0.24, *p* = .04). To verify if the correlation between the SN detection accuracy and ToM was not merely an experimental artifact, given the difficulty in detecting the emotionality in these Hellos, we recalculated the SN detection accuracies by removing each of the samples that was detected less than 50% of the time (*n* = 39); in fact, the strength of the correlation between the new SN detection accuracy and ToM increased (*r* = 0.37; *p* = .003; see Figure 2).^4^

As expected, there was no significant correlation between HS and HN detection accuracy and ToM (with HS, *r* = −0.16, two-tailed *p* = .23 and with HN, *r* = 0.12, two-tailed *p* = .37). Whereas SN or HN detection accuracy did not show an association with EQ (with SN, *r* = − 0.15, *p* = .14 and with HN, *r* = −0.005, two-tailed *p* = .97), unexpectedly, a low, but significant, *negative* correlation emerged between HS detection accuracy and EQ (*r* = −0.29, two-tailed *p* = .03); as empathy decreased, the tendency to detect differences between the happy and sad Hellos increased.

## Discussion

In the current study, we examined if third-party listeners could auditorily detect emotionality in women’s greetings directed to pictures of another woman displaying three different facial expressions of emotions—happy, sad, and neutral; these greetings were, on average, produced with predictable pitch patterns, and therefore, were considered as vocal responses mimicking the facial expressions. Whereas the sad Hellos and neutral Hellos differed from the happy Hellos in average pitch, the pitch of the sad and neutral Hellos were not significantly different from each other (Karthikeyan & Ramachandra, 2016). The detection accuracies, as expected, matched the average pitch differences: The happy Hellos could be detected at above-chance levels when these were presented along with sad and neutral Hellos in two separate forced choice listening tests (HS and HN tests, respectively). The sad and neutral Hellos could not be detected at above-chance levels (SN test). In line with the second hypothesis, we found a positive correlation between SN detection accuracy and ToM (but not EQ). An additional negative correlation emerged between HS detection accuracy and EQ, which was unexpected.

Although the negative (or any) correlation was unexpected between HS detection accuracy and EQ, on closer inspection, it is reminiscent of recent findings in autism research. We turn to this research because individuals with autism have been known to score low on EQ (Baron-Cohen & Wheelwright, 2004) and also exhibit hypersensitivity to pitch differences (Bonnel et al., 2003). Note that pitch discrimination ability predicts vocal emotion recognition ability (Globerson, Lavidor, Golan, Kishon-Rabin, & Amir, 2013). A correlation does not explain causation, but it is important to put these kinds of findings together in order to begin to address the negative correlation between EQ and HS detection accuracy.

Given that the happy and neutral Hellos, similar to the happy and sad Hellos, differed in average pitch, and that the HN and HS detection accuracy scores were not significantly different from each other, (*t*(55) = 1.43, *p* = .16), we could expect a negative association between HN detection accuracy and EQ similar to the negative correlation between HS detection accuracy and EQ, but such a correlation did not emerge. Again, research in autism, which has adopted forced choice tests similar to those used in the current study, has shown that these individuals are generally good at discriminating emotional expressions that are drastically different in emotional valence but are poor at discriminating subtle variations in emotions (Grossman & Tager-Flusberg, 2012). Unlike the happy and sad Hellos, the neutral and happy Hellos were only different in mean pitch but not in the variability of pitch, reducing the number of pitch-related cues (Karthikeyan & Ramachandra, 2016). Considering the facial expressions to which these Hellos were directed, as the changes in average pitch suggest, the happy and neutral faces differed mainly in arousal, and not in valence; the neutral face was judged to be of positive valence. Taken together, a negative correlation between empathy and vocal emotion detection accuracy may be observed only in cases where the pitch-relevant cues are hugely different, which can be expected for emotional expressions that vary in both valence and intensity.

Even though there was a positive correlation between SN detection accuracy and ToM, there was no correlation between SN detection accuracy and EQ. This is perhaps because of the potential dissociation of affective and cognitive empathy, as mentioned in the Introduction section, wherein affective empathy or the tendency to experience the feelings of others may not be necessary in mental state inferences made from a third-party’s perspective. Also, the EQ test, despite addressing some of the drawbacks of previous tests of empathy, may still not exclusively tap empathy (Bloom, 2016).

The association between ToM and SN detection accuracy in the current study is noteworthy. Among all the listening tests, the SN listening test emerged to be the most difficult, as the sad and neutral facial expressions, being different in valence alone, yielded response Hellos that were only subtly different from each other making these the most difficult to detect. Nevertheless, the detection accuracy of sad and neutral Hellos in the SN listening test correlated with the ToM scores.

It is relevant to note once again that the sad and neutral vocal responses were potential cues of empathy. As empathy has been shown to promote prosocial behavior (Stocks, Lishner, & Decker, 2009), being able to tune in auditorily (or through other channels) to the empathic tendencies of others would be beneficial. Consider, however, that the presence of an audience has been shown to enhance prosocial behavior (see review in Hamilton & Lind, 2016), which suggests that cooperative behavioral displays including emotional expressivity (Schug, Matsumoto, Horita, Yamagishi, & Bonnet, 2010) in the presence of observant others may not be indicative of the *actual* empathic tendencies.

As for the response Hellos used in the current study, these were not elicited in a real interaction while in the presence of others and hence cannot be expected to be as robustly emotional-sounding as these might have been; this is clear from the detection accuracies that ranged from about 50% to 57%. Whereas these numbers do not suggest that the listeners had a sensory or cognitive issue, these do indicate that while responding to emotional displays in the privacy of the testing room, speakers do not differentiate their greetings to the extent that these may be easily distinguished by third-party listeners. From an evolutionary perspective, however, detecting empathy cues in this kind of relatively *unmonitored* vocal behavior of others must have been important, and a tendency to eavesdrop on others’ interactions from a third-party’s stance must have contributed to the advanced mindreading ability exhibited by our species.

## Evolutionary Perspective on the Link Between Prosocial Displays and Eavesdropping and Its Potential Connection to Mindreading Ability

Prosocial displays increase with cues of vigilance (Fathi, Bateson, & Nettle, 2014; but see Northover, Pedersen, Cohen, & Andrews, 2016). Even young children tend to share more and steal less in the presence of observing peer than those that are not being observed (Engelmann, Herrmann, & Tomasello, 2012). If displays of prosocial behavior as a result of being watched, evolved, in part, as an adaptation that served reputation management in a complex social arena, a counter-adaptation in the form of a heightened tendency to eavesdrop can be expected, for it now becomes crucial to uncover the true prosocial tendencies of others (Locke, 2010).

Prior to the building of walls, examining an individual’s interactions from the vantage point of a third party largely unnoticed by that individual could have been achieved not just by hiding behind foliage but also by averting gaze and pretending indifference while tuning in auditorily. Gaze aversion has been discussed in a similar context as a way to secure privacy in open plan living arrangements in traditional societies (Locke, 2005, 2010). Given that even subtle signs of being looked at cause people to be more prosocial, looking away could also be a way of signaling inattention and thereby implicitly encouraging people in the vicinity to reveal more of their real selves in interactions, on which one can eavesdrop (largely) auditorily. In the absence of watchful eyes, however, only low-intensity prosocial signals can be expected (e.g., Manesi, Van Lange, & Pollet, 2016). This is reminiscent of the notion of “conspiratorial whispers” discussed by Krebs and Dawkins (1984)—weak signals narrowcast (and not broadcast) to a recipient. Our ancestors who tended to eavesdrop and be socially alert would have had a better and speedier chance of success in making the right decision about whom to approach (or not) in time of need as they would have gleaned the degree of “actual” prosociality exhibited by individuals in their social vicinity (see Trivers, 1971; also see Locke, 2005, 2010).^5^

In the current study, participating in the *Reading the Mind in the Eyes test* (the ToM test) and the third-party listening tests can be likened to the activity of eavesdropping. Like an eavesdropper who generally has access to relatively deteriorated information by virtue of the surreptitious nature of the activity,^6^ takers of these tests have access to incomplete information. As for the *Reading the Mind in the Eyes test*, which is known to be sensitive to individual differences in mindreading ability (Baron-Cohen et al., 2001), the test taker infers mental states from fragmented bits of visual information—expressions of the eye region; and in the listening tests employed in the current study, the listener, who was not involved in the original (simulated) dyadic event, has the opportunity to witness some aspects of that event—the facial expression of the woman and the emotion that is possibly embedded in the speaker’s Hello—but not all, as any potential visual cues of emotional mimicry by the speaker are unavailable.

Of these, the SN listening test, as discussed earlier, involved listening to nuanced vocal responses, where the *eavesdropping* participants would have had to strain to identify the responses, but these responses also happened to be potential cues of empathy (Karthikeyan & Ramachandra, 2016). Adopting an evolutionary analysis, it is not surprising that the ability to infer mental states based on degraded cues via the visual and auditory modes may be related, and that the need to eavesdrop may have facilitated the advancement of mindreading ability.

Taken together, long-term unrestrained prosociality would have been costly and evolutionarily unstable (e.g., Dawkins, 1976), which may have facilitated the strategic adjustment of self-presentational behaviors including emotional expressivity in public. This may have further set the stage for the need to examine others private selves, via eavesdropping,^7^ resulting in increasingly superior mechanisms for the detection of socially significant albeit weak signals.

In sum, the current study revealed that third-party listeners were not only able to detect those vocal responses that are different in average pitch but also their mindreading ability (measured via the *Reading the Mind in the Eyes test*) was associated with their ability to discern subtle variations in potentially empathic vocal responses. A future possibility is to examine vocal responses to positively and negatively valenced facial expressions of emotions in the presence and absence of others and to examine the perception of these responses by third-party listeners with respect to their mindreading ability.

## Notes

1 Note that the neutral face was judged to be of a positive valence (Karthikeyan & Ramachandra, 2016).

2 Although there were 41 speakers in the original study, the program randomly presented Hellos from only 40 speakers making the total stimuli equal to 80.

3 Box plots in SPSS identified three outliers in the detection accuracy scores; even with these removed, the findings remained the same (HS and HN detection accuracies were above chance, *p* < .05 and SN detection accuracy was at chance, *p* > .05). Also, note that one-tailed *p* values have been used for all directional hypotheses.

4 Two outliers that were more than 2 *SD* away from the newly calculated mean SN detection accuracy score were removed.

5 We should be cautious here to reasonably assume that these behavioral tendencies evolved in the environment of evolutionary adaptedness where living in small bands made it highly likely that members met and interacted with one another throughout life, which necessitated that prior and ongoing judgments were formed of everybody. In spite of the transformed social milieu of today where we increasingly find ourselves interacting with people whom we may not encounter more than once, eavesdropping can still be expected, even on interactions characterized by zero degree acquaintance.

6 Note that by eavesdropping we do not include instances of openly performed vigilance acts such as looking for predators; here, it is used in the restricted sense of tuning into others’ interactions (e.g., Locke, 2010).

7 Eavesdroppers and private selves refer to roles and not different populations. Any individual may adopt either role depending on the situation (e.g., Krebs & Dawkins, 1984). For the current study, we are discussing this in the context of third parties listening in for prosociality cues.
